# Randomised controlled trial of a supervised exercise rehabilitation program for colorectal cancer survivors immediately after chemotherapy: study protocol

**DOI:** 10.1186/1471-2407-7-154

**Published:** 2007-08-09

**Authors:** Rosalind R Spence, Kristiann C Heesch, Elizabeth G Eakin, Wendy J Brown

**Affiliations:** 1 School of Human Movement Studies, The University of Queensland, Brisbane, Australia; 2 School of Population Health, The University of Queensland, Brisbane, Australia

## Abstract

**Background:**

Colorectal cancer (CRC) diagnosis and the ensuing treatments can have a substantial impact on the physical and psychological health of survivors. As the number of CRC survivors increases, so too does the need to develop viable rehabilitation programs to help these survivors return to good health as quickly as possible. Exercise has the potential to address many of the adverse effects of CRC treatment; however, to date, the role of exercise in the rehabilitation of cancer patients immediately after the completion of treatment has received limited research attention. This paper presents the design of a randomised controlled trial which will evaluate the feasibility and efficacy of a 12-week supervised aerobic exercise program (ImPACT Program) on the physiological and psychological markers of rehabilitation, in addition to biomarkers of standard haematological outcomes and the IGF axis.

**Methods/Design:**

Forty CRC patients will be recruited through oncology clinics and randomised to an exercise group or a usual care control group. Baseline assessment will take place within 4 weeks of the patient completing adjuvant chemotherapy treatment. The exercise program for patients in the intervention group will commence a week after the baseline assessment. The program consists of three supervised moderate-intensity aerobic exercise sessions per week for 12 weeks. All participants will have assessments at baseline (0 wks), mid-intervention (6 wks), post-intervention (12 wks) and at a 6-week follow-up (18 wks). Outcome measures include cardio-respiratory fitness, biomarkers associated with health and survival, and indices of fatigue and quality of life. Process measures are participants' acceptability of, adherence to, and compliance with the exercise program, in addition to the safety of the program.

**Discussion:**

The results of this study will provide valuable insight into the role of supervised exercise in improving life after CRC. Additionally, process analyses will inform the feasibility of implementing the program in a population of CRC patients immediately after completing chemotherapy.

**Trial registration:**

ACTRN012606000395538

## Background

Although early detection of and treatment for colorectal cancer (CRC) have improved, the burden of this disease remains high. One million new cases of CRC were diagnosed worldwide in 2002, which was just under 10% of the world's total cancer incidence. In developed countries, where western lifestyles make the disease more common, incidence of CRC is second only to lung cancer [1].

Due to the relatively good overall prognosis for CRC patients, in 2002 approximately 351 000 people in developed countries were living with CRC within 5 years of diagnosis [1]. The majority of these patients underwent surgery and, depending on the characteristics of the tumour, chemotherapy and/or radiation. Although chemotherapy and radiation can greatly improve survival, they are toxic and often cause side effects that remain even after treatment is completed [2,3]. For these reasons CRC, as well as a number of other cancers, is increasingly becoming viewed as a chronic illness requiring longer-term management [4].

Although rehabilitation from cancer treatment continues until an individual recovers any major loss of function (a length of time which differs for every survivor), Courneya and Friedenreich [5] have suggested that the period from treatment completion to 3 to 6 months post-treatment be described as the 'rehabilitation' phase. As is the case for cardiac rehabilitation, the goal of rehabilitation after cancer treatment is to restore the person to good health as quickly as possible, which for cancer survivors means addressing the acute side effects resulting from the inherent toxicity of cancer treatment [5]. Common physiological and psychological side effects that remain after treatment include fatigue, reduced quality of life, decreased cardio-respiratory fitness, weight change, sleeping disorders, suppression of the immune system, and an increased risk of cancer recurrence and other chronic diseases [5,6]. These side effects can persist for months and even years following treatment completion; however, as the majority of the acute effects of treatment are concentrated within the first 3 to 6 months post-treatment, this is the time period in which survivors have the greatest need for rehabilitation [5]. Furthermore, many survivors report unanticipated fear and 'emptiness' after completing treatment, in part because they experience a relatively sharp down-turn in active medical and social support and they receive little, if any, information about what to expect following treatment completion [7].

Exercise has the potential to be an effective and feasible component of cancer rehabilitation. The rationale for the use of exercise programs in the rehabilitation of cancer survivors is two-fold. First, considerable evidence has accumulated over recent decades indicating that exercise and physical activity reduce symptoms and improve well-being among people with chronic diseases, such as diabetes, hypertension, coronary heart disease, chronic obstructive pulmonary disease, obesity, arthritis, and osteoporosis [8]. Given the successful use of exercise rehabilitation programs in addressing the symptoms of increased fatigue and reduced quality of life for cardiac patients, it is reasonable to expect that exercise would address these side effects in cancer patients. Second, there is a growing body of evidence, primarily from the breast cancer field, that suggests that exercise can play an important role in cancer management across the cancer experience, from diagnosis to survival [5,6,9-14]. Results from intervention studies suggest that exercise programs can have positive impacts on fatigue [15,16], quality of life [14], fitness [17], and immune function [18] among both cancer patients and long-term survivors. Moreover, evidence suggests that exercise is safe and feasible in cancer survivor populations [19].

During the past 20 years numerous cross-sectional and longitudinal have demonstrated that individuals who exercise regularly have a lower risk of developing CRC than sedentary individuals [11]. More recently, results from prospective observational studies have shown a protective association between physical activity or exercise after CRC diagnosis and survival [20,21].

Although evidence provides a strong rationale for the use of exercise in the rehabilitation of CRC patients, the question remains whether exercise interventions after CRC treatment will yield similar benefits to those seen with other chronic diseases and other cancer populations and whether a protective association between physical activity and CRC survival can be demonstrated through biologically plausible mechanisms monitored in an experimental intervention trial.

One biologically plausible hypothesis to explain the impact of exercise on CRC risk and prognosis is that exercise may decrease insulin-like growth factor (IGF) bioavailability via insulin-mediated changes in concentrations of insulin-like growth factor binding proteins (IGFBP) [22]. IGF-I plays a critical role in cellular proliferation and survival [22], and elevated levels have been associated with increased CRC risk [23]. In contrast, high pre-diagnosis levels of IGFBP-3 have been associated with prolonged survival after treatment [24]. If exercise programs can modulate the IGF axis, the risk of new primary cancers and cancer recurrence may be reduced. One study has provided evidence that exercise can impact biomarkers relevant to the IGF axis: Fairey et al [25] reported significant physiological changes in IGF-I, IGFBP-3 and IGF-I:IGFBP-3 molar ratio after 15 weeks of moderate-intensity exercise training in breast cancer survivors who were, on average, 14 months post-treatment. The clinical significance of these results and whether they can be replicated in CRC survivors is unknown. It is possible that similar changes could be evoked in a CRC population, and on the basis of the results of the observational studies, these changes could improve long-term prognosis.

Overall, evidence from previous studies suggests a strong rationale for offering exercise interventions in CRC survivors after treatment. However, the effectiveness of exercise on the 3- to 6-month rehabilitation of CRC survivors has not been investigated. For the general post-treatment time period (i.e. the broad period from treatment completion onwards), exercise intervention studies have predominately included breast cancer survivors [25-38] or mixed cancer populations [39-46]. Only one study of an exercise program after treatment exclusively recruited CRC survivors, but the intervention was not during the early 'rehabilitation' time period [47]. Although the intervention in that study was only two weeks in duration and the participants were heterogeneous in terms of the number of weeks since they completed treatment (from 4 – 40 weeks post-treatment), the exercise intervention was found to have a positive impact on CRC survivors' psychological and physiological health. To date only one previous study has focused specifically on rehabilitation in the time period immediately following treatment and that was with a breast cancer population. Hutnick and colleagues [27] offered a supervised exercise program commencing within 2–9 weeks of completion of breast cancer treatment and found that a moderate-intensity exercise program improved fitness and immune function and was both safe and feasible in a post-chemotherapy population.

Given that no study has evaluated the benefits of an exercise rehabilitation program for CRC survivors in the early rehabilitation period, there is much to be gained from evaluating the feasibility and efficacy of an exercise program in this population. Of particular need is a well designed, randomised controlled trial of CRC survivors. In light of the limited evidence supporting a beneficial role of exercise for CRC survivors immediately after treatment, the aim of this study is to investigate the effect of a supervised exercise program on the rehabilitation of CRC survivors immediately after treatment. As a phase one trial, the study will be tightly controlled for intervention parameters, such as exercise type, frequency, intensity and duration. Outcomes include changes in psychological and physiological markers of rehabilitation, in addition to biomarkers of standard haematological outcomes and the IGF axis. The process evaluation will focus on feasibility, recruitment, program safety, participant compliance with the intensity and duration of the exercise prescribed, and participant perceptions of the exercise program. The process evaluation is expected to yield information on factors that may increase or hamper the effectiveness of the program, information that may be crucial to the diffusion of the intervention if the program is found to be effective.

We hypothesize that the exercise program will be acceptable and safe for CRC survivors, that they will attend 90% of the exercise sessions and will comply with their exercise prescription. Additionally, we hypothesise that participants in the intervention group will have greater improvements in fitness, fatigue and quality of life and greater positive changes in IGF-1 and IGFBP-3 concentrations than those in a 'usual-care' control group. The results of this study will provide valuable new information about the role of exercise in improving CRC rehabilitation and survivorship.

## Methods/Design

### Overall aims of the study

To examine the effects of the ImPACT Program (I'm Physically Active after Cancer Treatment), a supervised exercise program commenced within 4 weeks of completing chemotherapy treatment, on psychological and physiological markers of rehabilitation in CRC survivors and to evaluate the feasibility of implementing such a program.

### Primary aims

#### Outcome Evaluation

1. To examine the effects of a supervised exercise program commenced within 4 weeks of completing chemotherapy treatment on cardio-respiratory fitness, biomarkers associated with health and survival, and indices of fatigue and quality of life in CRC survivors.

#### Process Evaluations

2. To assess the acceptability of the ImPACT Program to CRC survivors, their adherence to the exercise program, and their compliance with the prescribed duration and intensity during supervised exercise sessions.

3. To assess the safety of the exercise program by monitoring adverse events and changes in participants' haematological markers.

### Patient Recruitment

Forty patients who are due to complete adjuvant chemotherapy treatment for CRC within the duration of the study recruitment period will be recruited from oncology clinics in Brisbane, Queensland, Australia. Ethical approval for this study has been obtained from the participating hospitals as well as from The Medical Research Ethics Committee of The University of Queensland.

The treating oncologist will identify patients who are potentially eligible (patients undergoing adjuvant chemotherapy for CRC). A member of the clinic staff will then provide information about the program to these patients and seek permission for the program coordinator (RS) to make contact with the patients. The program coordinator will contact consenting patients to provide information about the study requirements and assess them for eligibility. Eligible patients who initially agree to join the program will be given a description of the program and, if still interested, required to give written informed consent prior to baseline testing, which will occur within 4 weeks of treatment completion.

Patients will be recruited and will start the program on a 'rolling' basis, with an estimated 5 patients recruited per month for 9 months (based on the current rate of patient completion at the recruiting clinic). Forty patients are expected to have been recruited and to have completed involvement in the study within a 12-month period. It is expected that results will be reported within 12 months of the completion of the study.

#### Patient inclusion criteria

a. Aged between 18 and 75 years, with confirmed CRC (stage I-III).

b. Treated for CRC with surgery before then completing adjuvant chemotherapy treatment within the 4 weeks prior to enrolment in the study.

c. Non-smokers (not having smoked during the previous 12 months).

d. Able to read, write and understand English.

e. Willing and able to attend supervised exercise sessions 3 times a week for a period of 12 weeks, with an intention of achieving a 90% attendance.

#### Patient exclusion criteria

a. Metastatic or incurable CRC.

b. Physical/psychiatric impairments that would seriously impair physical mobility.

c. Known contraindications for exercise (as assessed by a pre-screening questionnaire modified from the Sports Medicine Australia Pre-Exercise Screening System 2005 [48]).

### Study Design

**Figure 1:**
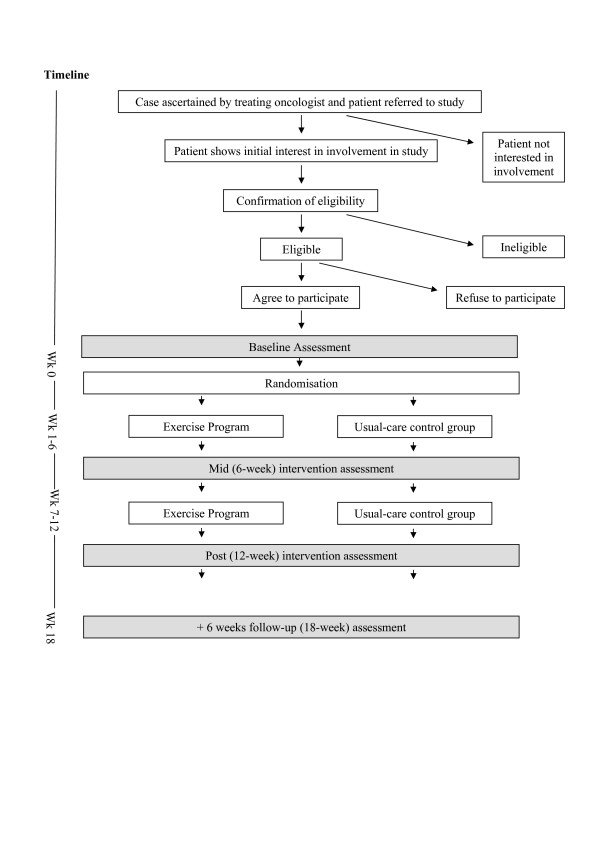
Flow of participants through study.

This study is designed as a two-group randomised controlled trial (see figure 1 for flow of participants). After baseline testing, patients will be allocated to either an intervention group that receives the exercise program (EX) or a control group that receives 'usual-care' (CG). The exercise program will last 12 weeks and will start within one week of baseline testing. To reduce drop-out of CG participants, they will be offered an exercise program after completing the final study assessment. Outcome measures will be assessed at baseline, at the mid-intervention time point of 6 weeks, in the week after the intervention period and 6 weeks after the intervention is completed (weeks 0, 6, 12 and 18).

### Randomisation

To ensure that the study groups are balanced for potentially confounding variables, a dynamic form of randomisation called 'minimisation' [49,50] will be used to allocate patients to either EX or CG. Confounding variables for which the groups will be balanced are: age (< 50 years of age or ≥ 50 years of age), sex, and self-report physical activity history ('never regularly physically active' or 'regularly physically active before and/or during treatment'). Randomisation will be performed by a computer program designed for this purpose [51]. Participants will be randomised at the completion of the baseline testing.

### Power calculation and sample size

Fatigue was chosen as the primary outcome for computing sample size. Data used in the computation are from an intervention with breast cancer survivors, as very little research has been conducted with CRC survivors. Courneya et al [32] reported a clinically significant decrease of 9.3 (± 10.2) in fatigue scores (as measured by the FACT-fatigue scale, the same tool to be used in this study), between baseline and the completion of a 15-week intervention, among participants enrolled in a supervised exercise program. A sample of 14 participants per group will allow us to detect a change of this magnitude (i.e. 14% difference) in FACT scores pre- to post-intervention and between groups post-intervention, with a power of 80% and an alpha level of 0.05. Assuming a 30% drop out rate, we will need to recruit 40 participants, 20 in each group, to allow us to detect these differences in fatigue.

### Intervention

Participants randomised to EX will complete an aerobic exercise session on three days of the week for 12 weeks. All sessions will be completed under the guidance and supervision of an exercise physiologist. These sessions will be scheduled for a time and location that is mutually convenient to the participant and exercise physiologist.

EX participants will commence the program with sessions of 20 minutes in duration (plus 5-minute warm-up and 5-minutes cool-down at a light intensity) and with the guidance of the exercise physiologist, will increase by week 10 to a duration of 40 minutes (plus warm-up and cool-down). Similarly, exercise sessions will commence at a 'moderate' intensity (approximating 40–50% of heart rate reserve or a Rating of Perceived Exertion [RPE] of 11–12) and will increase to a 'high' intensity (approximating 70–80% of heart rate reserve or a RPE of 15–16) as the participant is able over the 12-week exercise program. Although the exercise 'goal' (i.e. to complete sessions of 40 minutes at 'high' intensity) is identical for all participants, the exercise prescription for each session will be individually tailored to each participant. Therefore, some participants may reach their exercise 'goal' earlier than others. The framework for progression of the prescribed exercise intensity and duration will be based on ACSM's exercise prescription guidelines for both general [52] and chronic disease populations [53].

The exercise physiologist will reassess and progress each EX participant's exercise prescription throughout sessions, increasing the duration and intensity of the sessions as the participant is willing and able. Both heart rate and RPE will be used concurrently and as appropriate for each participant. Exercise prescription is recognised as an 'art' which involves the combination of objective and subjective indices [52]. The concurrent use of heart rate and perceived exertion more accurately achieves targeted work rates than either measure alone, and RPE alone has been shown to be effective in achieving a training effect that does not differ significantly from that of heart rate alone [54].

Heart rate will be recorded throughout the session by a heart rate monitor (Polar s610i, Polar Electro Oy, Finland) at 5-second epochs, and the participant's self-reported RPE will be recorded by the exercise physiologist at 5-minute intervals.

### Control Group

The CG will receive 'usual-care' during the course of the study. Usual care after adjuvant chemotherapy for CRC patients is generally a single follow-up visit with the treating oncologist 6 months after completion of treatment. From baseline testing until the final assessment at the 18-week follow-up, CG participants will be instructed to maintain their current physical activity level. To reduce attrition, CG participants will be offered the exercise program after the 18-week follow-up assessment.

### Outcome measures

The exercise program will last for 12 weeks, with all outcomes assessed at baseline, mid-intervention, post-intervention, and 6-week follow-up (0, 6, 12 and 18 weeks, see table 1) unless otherwise stated. A summary of the tool to be used for each outcome is described in table 2.

**Table 1:** Outcome variables for effect evaluation

**Variable**	**No. of items**	**Baseline**	**mid-int**	**post-int**	**fw**
**Primary and Secondary outcomes**					

Fatigue (FACIT-fatigue) [60]	13	Q	Q	Q	Q
QoL standard (SF-36) [62]	36	Q	Q	Q	Q
*physical functioning*	10	Q	Q	Q	Q
*social functioning*	2	Q	Q	Q	Q
*role limitations – physical*	4	Q	Q	Q	Q
*role limitations – emotional*	3	Q	Q	Q	Q
*general medical health*	5	Q	Q	Q	Q
*bodily pain*	2	Q	Q	Q	Q
*vitality*	4	Q	Q	Q	Q
*mental health*	5	Q	Q	Q	Q
*general medical perceptions*	1	Q	Q	Q	Q
QoL cancer specific (EORTC QLQ-C30) [63]	30	Q	Q	Q	Q
*physical*	5	Q	Q	Q	Q
*role*	2	Q	Q	Q	Q
*cognitive*	2	Q	Q	Q	Q
*emotional*	4	Q	Q	Q	Q
*social*	2	Q	Q	Q	Q
*fatigue*	3	Q	Q	Q	Q
*pain*	2	Q	Q	Q	Q
*nausea and vomiting*	2	Q	Q	Q	Q
*global health and QoL*	2	Q	Q	Q	Q
Physical Fitness					
*MET value for final stage completed and heart rate at completion of second stage (Modified Bruce Submaximal Treadmill Protocol)*	N/A	EP	EP	EP	EP
*Field Test (6-minute Walk Test)*	N/A	EP	EP	EP	EP
Haematological Markers					
*Full blood count*	N/A	P	P	P	P
*Full iron studies*	N/A	P	P	P	P
*IGF-1*	N/A	P	P	P	P
*IGFBP-3*	N/A	P	P	P	P
**Additional Measures**					
Time since diagnosis	1	Q + MR			
Stage of disease	1	Q + MR			
Socio-demographic variables	10	Q			
Physical Activity Behaviour					
*Self-report recall (Active Australia + history question)*	18 + 2	Q+T	T (no history)	T (no history)	T (no history)
*PA Logbook*	24	LOG	LOG	LOG	LOG
BMI	2	EP	EP	EP	EP
WHR	2	EP	EP	EP	EP

Q = written questionnaire, EP = Exercise Physiologist (RS), LOG = self-report logbook, T = telephone-delivered questionnaire, P = pathology lab, MR = Medical Records, QoL = Quality of Life, BMI = Body Mass Index, WHR = Waist Hip Ratio

**Table 2:** Outcome variables for process evaluation

**Process evaluation outcome variables**	**Before/during intervention**	**Follow-up**
**Attendance by patients**		

Number of sessions attended by each patient	EP record	-
Reason for stopping during the intervention	Questionnaire	-
**Adherence by patient**		
Exercise intensity	RPE EP record	-
	HR monitor download	-
Exercise duration: minutes	EP record	-
	HR monitor download	-
**Safety**		
Self report adverse effects (injury etc)	EP record	-
Full blood count	Blood test	Blood test
Anaemia (full iron studies)	Blood test	Blood test
**Opinion about the program (EX only)**		
Enjoyment of the program	-	Questionnaire
Feelings about attending sessions	-	Questionnaire
Reasons for missed sessions	-	Questionnaire
Timing of the program	-	Questionnaire
Benefits from the program	-	Questionnaire
Plans to continue exercising	-	Questionnaire
Favourite/least favourite aspects of the program	-	Questionnaire
Recommending the program to other patients	-	Questionnaire
Recommendations for improvements	-	Questionnaire
Other comments about the program	-	Questionnaire

EP = Exercise Physiologist (RS), EX = Exercise group, HR = Heart Rate, RPE = Rating of Perceived Exertion

### Cardio-respiratory fitness

Cardio-respiratory fitness will be indirectly assessed using the Modified Bruce Treadmill protocol, which is a submaximal incremental exercise test [52]. Treadmill protocols are popular in clinical testing because walking is a familiar activity and uses a large muscle mass [55]. The Modified Bruce Protocol is similar to the widely used Bruce protocol; however, the treadmill is initially horizontal rather than uphill, with the slope increasing to a 5%, 10% and 12% gradient in the 2^nd^, 3^rd ^and 4^th ^stages respectively. Additionally, the first three stages are conducted at a constant speed of 2.7 km/hr and with only the 4^th ^stage increasing in speed (to 4 km/hr) [52]. These modifications in protocol are important for older and deconditioned populations, as the modifications increase the likelihood of participants reaching an optimal test duration of 8–12 minutes [56] before volitional fatigue or heart rate targets require test termination (test terminates when heart rate = 85% of [220 – age]).

Clinical populations often do not meet the 'steady-state' heart rate requirement of VO2 prediction from submaximal exercise testing [55]. For this reason, the results of the treadmill test will be reported as heart rate at the end of the second stage of the test, as well as the estimated oxygen cost (ie metabolic equivalent [METs]) for the last stage completed [57]. Additionally, to ensure that comprehensive fitness data are available for all participants, the 6-minute walk test [58] will be performed by each participant after attempting the Modified Bruce Protocol. Participants will have a rest of 15 minutes between these tests, during which time they will complete written components of the assessment. The 6-minute walk test is a validated, simple and safe test, often used with chronic heart failure and chronic obstructive pulmonary disease patients to assess the effects of a rehabilitation program [59].

### Biomarkers of health

Within 3 days of each assessment, participants will attend a convenient, accredited pathology laboratory where blood will be drawn from an antecubital vein by qualified staff. To minimise acute responses and diurnal fluctuations, blood tests will occur at least 48 hours after exercise and between 7:30–10:00 am (participants will be asked to schedule all their tests for approximately the same time of day). All blood tests will be preceded by an overnight (12 hour) fast.

IGF-1 and IGFBP-3 will be measured at each assessment time point. All analyses will be performed by one pathology laboratory. Blood samples will also be stored at -70°C for further analyses at a later date, if funding allows.

### Fatigue

Fatigue will be assessed by the 13-item Fatigue Scale (FACT-FS) of the FACIT measurement system [60]. The FACT-FS was developed specifically for the cancer population and has been shown to be valid and reliable in the general cancer population [60].

### Quality of life

As in a similar study by Adamsen et al [61], two quality of life (QoL) questionnaires, the SF-36 [62] and the EORTC QLQ-C30 [63], will be used to comprehensively cover the different aspects of physical and psychosocial well-being.

The two instruments assess QoL at slightly different time periods, with the SF-36 including questions about the current period (15 items), the last 4 weeks (20 items) and the past year (1 item). The EORTC QLQ-C30 focusses on the current period (5 items) and the past week (25 items).

The 36-item SF-36 is a frequently used measure of well-being in the general population, and it has been shown to be valid and reliable in cancer populations [64]. It contains eight subscales measuring general health concepts and one global health question. Five of the subscales (physical functioning, role limitations – physical, role limitations – emotional, social functioning, and bodily pain) measure degree of dysfunction, and three (general health perceptions, vitality, and mental health) measure the full range from negative to positive health conditions.

The 30-item EORTC QLQ-C30 was developed specifically to measure health-related quality of life in cancer patients and includes assessment of symptoms and side effects. Six functional subscales (physical, role, cognitive, emotional, social functioning, and global QoL) and eight symptom subscales (fatigue, nausea/vomiting, pain, dyspnoea, insomnia, appetite loss, constipation, and diarrhoea) comprise the EORTC QLQ-C30 questionnaire. The EORTC QLQ-C30 has been shown to have good validity and reliability properties [63,65].

### Feasibility

#### Program Acceptability

Participant feedback about the ImPACT program will be collected using a structured written evaluation form at the post-intervention (12-week) assessment. EX participants will be asked how enjoyable they found the ImPACT Program (five-point scale: "*extremely enjoyable*"- "*not at all enjoyable*") and how they felt about attending the ImPACT Program (five-point scale: "*I looked forward to it all the time*"- "*I did not look forward to it at all*"). In addition, EX participants will be asked open-ended questions about the most and least enjoyable aspects of the ImPACT Program, any improvements they noticed in themselves since commencing the program, why they missed sessions, their opinion of the timing of the program with regard to treatment, whether they would recommend it to other cancer survivors, and whether they intend to continue exercising. Finally, participants will be asked if they would have preferred that the ImPACT Program had been 1) a group-based, 2) an exercise program to do on their own, 3) as offered – they liked the program as it was, or 4) OTHER (asked to specify). They will be given the opportunity to write any other comments about their experiences in or perceptions of the program.

#### Adherence and Compliance

Adherence to the ImPACT Program will be defined as EX participants' attendance at the exercise sessions, whereas compliance will be defined as how closely they follow their individualised exercise prescription for duration and intensity at each exercise session. Attendance will be monitored by the supervising exercise physiologist. Compliance with the prescribed program will be measured subjectively using RPE and objectively by the downloadable heart rate monitors. Every 5 minutes the supervising exercise physiologist will ask participants to state their RPE, which will be recorded on the supervisor's record sheet.

#### Safety

An adverse event will be defined as any adverse change from a participant's baseline condition, regardless of whether it is considered related to exercise training [32]. In addition to participant self-report of adverse effects on an ad hoc basis, haematological parameters will be assessed at 0, 6, 12 and 18 weeks. Analysis of full blood count and full iron studies will allow monitoring of participants' general health and identification of any participants whose health would be compromised by continued involvement. Results of the full iron studies will also be used to ensure that participants are not anaemic, as anaemia could confound self-report fatigue scores [66]. These analyses will also provide the opportunity to identify any differences in haematological parameters between study groups. The protocol for these analyses is the same as previously described for the biomarkers.

### Additional Measures

Additional variables will be assessed to gain a better understanding of the participants and to allow for a comparison between groups. Socio-demographic characteristics (gender, age, marital status, living arrangements, education level and occupation) will be measured by self-report at baseline. Health-related variables (eg disease status, time since diagnosis, type of treatment, time since treatment and other chronic conditions) will be assessed at baseline by self-report and, if consent is given, confirmed by a review of medical records. Descriptive characteristics (weight, height, waist and hip circumference) will be measured at each assessment and used to calculate body mass index and waist-hip ratio.

### Physical Activity

Although not a primary outcome, physical activity behaviour will be assessed at baseline, mid-intervention, post-intervention and 6-week follow-up. These data will be used to compare physical activity levels between the study groups and identify potentially contaminating levels of physical activity external to the intervention. A physical activity logbook, detailing sedentary, light, moderate and high intensity physical activity lasting at least 10 minutes per bout during the previous week, will be completed by all participants for the week after each of the four assessments. Diaries and logbooks require intensive effort from participants, but the data collected are comprehensive and represent the only way to measure all forms of activity, including transport, work, home and leisure-time activity [67]. In case there is poor compliance with the diaries, physical activity will also be measured with the validated Active Australia questionnaire [68-70], administered by telephone the week after each assessment.

Additionally, supplementary questions, developed by the researchers based on physical activity categories described by Hayes et al [71], will be asked to investigate participants' recall of their physical activity level both in the year before their cancer diagnosis and during cancer treatment ("*no physsical activity", "sporadic or limited physical activity", "regular physical activity"*). These supplementary questions will be asked in the baseline questionnaire and then again a week later during the administration of the Active Australia survey, to evaluate the test-retest reliability of the questions.

### Data Analysis

Descriptive techniques will be used to characterise study participants. Data collected for the impact evaluation will be analysed according to intention-to-treat and per-protocol principles. Specifically, differences between EX and CG in changes in the outcome variables between baseline and the 6-, 12- and 18-week assessments will be analysed using linear regression models. EX participants will be included in the per-protocol analysis if they attend 90% of the exercise sessions. Data collected for the process evaluation will be analysed using both quantitative and qualitative techniques.

## Discussion

As improvements in CRC detection and treatment continue to extend the lives of CRC patients, increased attention has been given to improving the lives of survivors. Although exercise interventions have shown some promise in attenuating treatment side-effects in other cancer populations, no study to-date has looked at the benefit of a supervised exercise program in the rehabilitation of CRC survivors immediately following the completion of adjuvant treatment. The results of this study will provide valuable insight into the role that a supervised exercise program could play in improving quality of life after CRC.

## Competing interests

The author(s) declare that they have no competing interests.

## Authors' contributions

RS developed the idea for the study. RS, WB, KH and EE were involved in further developing the protocol. RS was responsible for drafting the manuscript and will implement the protocol and collect the data. All authors contributed to the final manuscript.

## Pre-publication history

The pre-publication history for this paper can be accessed here:



## References

[B1] Parkin DM, Bray F, Ferlay J, Pisani P (2005). Global cancer statistics, 2002. CA Cancer J Clin.

[B2] Baker F, Denniston M, Smith T, West MM (2005). Adult cancer survivors: how are they faring?. Cancer.

[B3] Jansman FG, Sleijfer DT, de Graaf JC, Coenen JL, Brouwers JR (2001). Management of chemotherapy-induced adverse effects in the treatment of colorectal cancer. Drug Saf.

[B4] Pinto BM, Trunzo JJ (2005). Health behaviors during and after a cancer diagnosis. Cancer.

[B5] Courneya KS, Friedenreich CM (2001). Framework PEACE: an organizational model for examining physical exercise across the cancer experience. Ann Behav Med.

[B6] Courneya KS (2003). Exercise in cancer survivors: an overview of research. Med Sci Sports Exerc.

[B7] Stanton AL, Ganz PA, Rowland JH, Meyerowitz BE, Krupnick JL, Sears SR (2005). Promoting adjustment after treatment for cancer. Cancer.

[B8] Pedersen BK, Saltin B (2006). Evidence for prescribing exercise as therapy in chronic disease. Scand J Med Sci Sports.

[B9] Galvao DA, Newton RU (2005). Review of exercise intervention studies in cancer patients. J Clin Oncol.

[B10] Irwin ML, Ainsworth BE (2004). Physical activity interventions following cancer diagnosis: methodologic challenges to delivery and assessment. Cancer Invest.

[B11] Friedenreich CM, Orenstein MR (2002). Physical activity and cancer prevention: etiologic evidence and biological mechanisms. J Nutr.

[B12] Friedenreich CM (2001). Physical activity and cancer prevention: from observational to intervention research. Cancer Epidemiol Biomarkers Prev.

[B13] Courneya KS, Mackey JR, Jones LW (2000). Coping with cancer: can exercise help? (Comment faire face au cancer.). Physician and sportsmedicine (New York).

[B14] Courneya KS, Friedenreich CM (1999). Physical exercise and quality of life following cancer diagnosis: a literature review. Ann Behav Med.

[B15] Stricker CT, Drake D, Hoyer KA, Mock V (2004). Evidence-based practice for fatigue management in adults with cancer: exercise as an intervention. Oncol Nurs Forum.

[B16] Lucia A, Earnest C, Perez M (2003). Cancer-related fatigue: can exercise physiology assist oncologists?. Lancet Oncol.

[B17] McTiernan A (2004). Physical activity after cancer: physiologic outcomes. Cancer Invest.

[B18] Fairey AS, Courneya KS, Field CJ, Mackey JR (2002). Physical exercise and immune system function in cancer survivors: a comprehensive review and future directions. Cancer.

[B19] Schmitz KH, Holtzman J, Courneya KS, Masse LC, Duval S, Kane R (2005). Controlled physical activity trials in cancer survivors: a systematic review and meta-analysis. Cancer Epidemiol Biomarkers Prev.

[B20] Meyerhardt JA, Giovannucci EL, Holmes MD, Chan AT, Chan JA, Colditz GA, Fuchs CS (2006). Physical activity and survival after colorectal cancer diagnosis. J Clin Oncol.

[B21] Meyerhardt JA, Heseltine D, Niedzwiecki D, Hollis D, Saltz LB, Mayer RJ, Thomas J, Nelson H, Whittom R, Hantel A, Schilsky RL, Fuchs CS (2006). Impact of physical activity on cancer recurrence and survival in patients with stage III colon cancer: findings from CALGB 89803. J Clin Oncol.

[B22] Giovannucci E (2001). Insulin, insulin-like growth factors and colon cancer: a review of the evidence. J Nutr.

[B23] Sandhu MS, Dunger DB, Giovannucci EL (2002). Insulin, insulin-like growth factor-I (IGF-I), IGF binding proteins, their biologic interactions, and colorectal cancer. J Natl Cancer Inst.

[B24] Haydon AM, Macinnis RJ, English DR, Morris H, Giles GG (2006). Physical activity, insulin-like growth factor 1, insulin-like growth factor binding protein 3, and survival from colorectal cancer. Gut.

[B25] Fairey AS, Courneya KS, Field CJ, Bell GJ, Jones LW, Mackey JR (2003). Effects of exercise training on fasting insulin, insulin resistance, insulin-like growth factors, and insulin-like growth factor binding proteins in postmenopausal breast cancer survivors: a randomized controlled trial. Cancer Epidemiol Biomarkers Prev.

[B26] Matthews CE, Wilcox S, Hanby CL, Der Ananian C, Heiney SP, Gebretsadik T, Shintani A (2006). Evaluation of a 12-week home-based walking intervention for breast cancer survivors. Support Care Cancer.

[B27] Hutnick NA, Williams NI, Kraemer WJ, Orsega-Smith E, Dixon RH, Bleznak AD, Mastro AM (2005). Exercise and lymphocyte activation following chemotherapy for breast cancer. Med Sci Sports Exerc.

[B28] Schmitz KH, Ahmed RL, Hannan PJ, Yee D (2005). Safety and efficacy of weight training in recent breast cancer survivors to alter body composition, insulin, and insulin-like growth factor axis proteins. Cancer Epidemiol Biomarkers Prev.

[B29] Fairey AS, Courneya KS, Field CJ, Bell GJ, Jones LW, Mackey JR (2005). Randomized controlled trial of exercise and blood immune function in postmenopausal breast cancer survivors. J Appl Physiol.

[B30] Fairey AS, Courneya KS, Field CJ, Bell GJ, Jones LW, Martin BS, Mackey JR (2005). Effect of exercise training on C-reactive protein in postmenopausal breast cancer survivors: A randomized controlled trial. Brain Behav Immun.

[B31] Turner J, Hayes S, Reul-Hirche H (2004). Improving the physical status and quality of life of women treated for breast cancer: a pilot study of a structured exercise intervention. J Surg Oncol.

[B32] Courneya KS, Mackey JR, Bell GJ, Jones LW, Field CJ, Fairey AS (2003). Randomized controlled trial of exercise training in postmenopausal breast cancer survivors: cardiopulmonary and quality of life outcomes. J Clin Oncol.

[B33] McKenzie DC, Kalda AL (2003). Effect of upper extremity exercise on secondary lymphedema in breast cancer patients: a pilot study. J Clin Oncol.

[B34] McTiernan A, Ulrich C, Kumai C, Bean D, Schwartz R, Mahloch J, Hastings R, Gralow J, Potter JD (1998). Anthropometric and hormone effects of an eight-week exercise-diet intervention in breast cancer patients: results of a pilot study. Cancer Epidemiol Biomarkers Prev.

[B35] Segar ML, Katch VL, Roth RS, Garcia AW, Portner TI, Glickman SG, Haslanger S, Wilkins EG (1998). The effect of aerobic exercise on self-esteem and depressive and anxiety symptoms among breast cancer survivors. Oncol Nurs Forum.

[B36] Nieman DC, Cook VD, Henson DA, Suttles J, Rejeski WJ, Ribisl PM, Fagoaga OR, Nehlsen-Cannarella SL (1995). Moderate exercise training and natural killer cell cytotoxic activity in breast cancer patients. Int J Sports Med.

[B37] Peters C, Lotzerich H, Niemeier B, Schule K, Uhlenbruck G (1994). Influence of a moderate exercise training on natural killer cytotoxicity and personality traits in cancer patients. Anticancer Res.

[B38] Peters C, Lotzerich H, Niemeir B, Schule K, Uhlenbruck G (1995). Exercise, cancer and the immune response of monocytes. Anticancer Res.

[B39] Oldervoll LM, Kaasa S, Knobel H, Loge JH (2003). Exercise reduces fatigue in chronic fatigued Hodgkins disease survivors--results from a pilot study. Eur J Cancer.

[B40] Burnham TR, Wilcox A (2002). Effects of exercise on physiological and psychological variables in cancer survivors. Med Sci Sports Exerc.

[B41] Na YM, Kim MY, Kim YK, Ha YR, Yoon DS (2000). Exercise therapy effect on natural killer cell cytotoxic activity in stomach cancer patients after curative surgery. Arch Phys Med Rehabil.

[B42] Porock D, Kristjanson LJ, Tinnelly K, Duke T, Blight J (2000). An exercise intervention for advanced cancer patients experiencing fatigue: a pilot study. J Palliat Care.

[B43] Durak EP, Lilly PC (1998). The application of an exercise and wellness program for cancer patients: A preliminary outcome report. Journal of strength and conditioning research.

[B44] Dimeo FC, Tilmann MH, Bertz H, Kanz L, Mertelsmann R, Keul J (1997). Aerobic exercise in the rehabilitation of cancer patients after high dose chemotherapy and autologous peripheral stem cell transplantation. Cancer.

[B45] Sharkey AM, Carey AB, Heise CT, Barber G (1993). Cardiac rehabilitation after cancer therapy in children and young adults. Am J Cardiol.

[B46] Winningham ML, Watson RR, Eisinger M (1992). The role of exercise in cancer therapy. Exercise and Disease.

[B47] Allgayer H, Nicolaus S, Schreiber S (2004). Decreased interleukin-1 receptor antagonist response following moderate exercise in patients with colorectal carcinoma after primary treatment. Cancer Detect Prev.

[B48] Norton K, Australian Government - Department of Health and Aging, Sports Medicine Australia (2005). Sports Medicine Australia (SMA) pre-exercise screening system 2005.

[B49] Treasure T, MacRae KD (1998). Minimisation: the platinum standard for trials?. Randomisation doesn't guarantee similarity of groups; minimisation does. Bmj.

[B50] Saxton JM, Daley A, Woodroofe N, Coleman R, Powers H, Mutrie N, Siddall V, Crank H (2006). Study protocol to investigate the effect of a lifestyle intervention on body weight, psychological health status and risk factors associated with disease recurrence in women recovering from breast cancer treatment [ISRCTN08045231]. BMC Cancer.

[B51] Evans SJ, Day SJ, Royston P (1990). Minimisation Program for Allocating Patients to Treatments in Clinical Trials.

[B52] Whaley MH, Brubaker PH, Otto RM, Armstrong LE, American College of Sports Medicine., American College of Sports Medicine. (2006). ACSM's guidelines for exercise testing and prescription.

[B53] American College of Sports Medicine. (2003). ACSM's exercise management for persons with chronic diseases and disabilities.

[B54] Ilarraza H, Myers J, Kottman W, Rickli H, Dubach P (2004). An evaluation of training responses using self-regulation in a residential rehabilitation program. J Cardiopulm Rehabil.

[B55] Lear SA, Brozic A, Myers JN, Ignaszewski A (1999). Exercise stress testing. An overview of current guidelines. Sports Med.

[B56] Myers J, Froelicher VF (1993). Exercise testing. Procedures and implementation. Cardiol Clin.

[B57] Warburton DE, Nicol CW, Bredin SS (2006). Prescribing exercise as preventive therapy. Cmaj.

[B58] American Thoracic Society (2002). ATS statement: guidelines for the six-minute walk test. Am J Respir Crit Care Med.

[B59] Kervio G, Carre F, Ville NS (2003). Reliability and intensity of the six-minute walk test in healthy elderly subjects. Med Sci Sports Exerc.

[B60] Yellen SB, Cella DF, Webster K, Blendowski C, Kaplan E (1997). Measuring fatigue and other anemia-related symptoms with the Functional Assessment of Cancer Therapy (FACT) measurement system. J Pain Symptom Manage.

[B61] Adamsen L, Quist M, Midtgaard J, Andersen C, Moller T, Knutsen L, Tveteras A, Rorth M (2006). The effect of a multidimensional exercise intervention on physical capacity, well-being and quality of life in cancer patients undergoing chemotherapy. Support Care Cancer.

[B62] Ware JE, Sherbourne CD (1992). The MOS 36-item short-form health survey (SF-36). I. Conceptual framework and item selection. Med Care.

[B63] Aaronson NK, Ahmedzai S, Bergman B, Bullinger M, Cull A, Duez NJ, Filiberti A, Flechtner H, Fleishman SB, de Haes JC (1993). The European Organization for Research and Treatment of Cancer QLQ-C30: a quality-of-life instrument for use in international clinical trials in oncology. J Natl Cancer Inst.

[B64] Pinar R (2005). Reliability and construct validity of the SF-36 in Turkish cancer patients. Qual Life Res.

[B65] Osoba D, Aaronson N, Zee B, Sprangers M, te Velde A (1997). Modification of the EORTC QLQ-C30 (version 2.0) based on content validity and reliability testing in large samples of patients with cancer. The Study Group on Quality of Life of the EORTC and the Symptom Control and Quality of Life Committees of the NCI of Canada Clinical Trials Group. Qual Life Res.

[B66] Patterson AJ, Brown WJ, Powers JR, Roberts DC (2000). Iron deficiency, general health and fatigue: results from the Australian Longitudinal Study on Women's Health. Qual Life Res.

[B67] U.S. Department of Health and Human Services (1996). Physical activity and health : a report of the Surgeon General.

[B68] Brown WJ, Trost SG, Bauman A, Mummery K, Owen N (2004). Test-retest reliability of four physical activity measures used in population surveys. J Sci Med Sport.

[B69] Timperio A, Salmon J, Rosenberg M, Bull FC (2004). Do logbooks influence recall of physical activity in validation studies?. Med Sci Sports Exerc.

[B70] Brown W, Bauman A, Chey T, Trost S, Mummery K (2004). Comparison of surveys used to measure physical activity. Aust N Z J Public Health.

[B71] Hayes SC, Rowbottom D, Davies PS, Parker TW, Bashford J (2003). Immunological changes after cancer treatment and participation in an exercise program. Med Sci Sports Exerc.

